# Empowering future nurses: a focus group study on simulation for transition to clinical practice

**DOI:** 10.1186/s12912-025-04229-9

**Published:** 2025-12-16

**Authors:** Kristine Haddeland, Hanne Synøve Briseid, Hege Kristin Aslaksen Kaldheim, Hege Mari Johnsen

**Affiliations:** Faculty of Health and Sport Sciences, University of Agder, Post box 422, Kristiansand, 4604 Norway

**Keywords:** Nursing education, Simulation training, Quality indicators, Qualitative research

## Abstract

**Background:**

Simulation exercises are increasingly being used in undergraduate nursing education. The aim of this study was to identify, describe and discuss the factors that facilitate the successful implementation of simulation exercises for the transition to clinical practice in primary healthcare settings experienced by nursing students.

**Methods:**

Seven focus group interviews were conducted with a total of 35 undergraduate nursing students from two university campuses in Norway. There were three focus groups with first-year students, two focus groups with second-year students and two focus groups with third-year students. The interviews were analysed using reflexive thematic analysis.

**Results:**

The analysis identified four main themes, each including essential factors for successful implementation of simulation exercises: (1) feeling secure (2), experiencing realism (3), learning in different roles and (4) reflecting together.

**Conclusions:**

The findings outline key factors experienced by the participants that may contribute to successful simulations. These insights may guide future implementation of simulation exercises in undergraduate nursing education, specifically for the transition to clinical practice in primary healthcare settings.

**Clinical trial number:**

Not applicable.

## Background

Nursing programmes are under increasing pressure to graduate more nursing students [1]. With more students needing clinical placements, increasing pressure is placed on universities to offer quality clinical experiences and clinical placement sites [2]. According to the European Union standards and Norwegian nursing curriculum, approximately 50% (90 ECTS) of undergraduate nursing education should comprise placements in the clinical field as learning environments. In Norway, several models are used to organize clinical placements [3]. The most common supervision model involves three primary participants: a nursing student, a nurse preceptor, and a nurse educator. The nurse preceptor is a registered nurse working in the clinical field, such as a nursing home or hospital, while the nurse educator is employed by an educational institution like a university or university college. The nurse preceptors’ role includes providing daily face-to-face guidance, follow-up, supervision, and evaluation of the nursing student. On the other hand, the nurse educator ensures the clinical practice period affords the nursing student an optimal learning experience and provides a fair assessment of the learning outcomes achieved [4]. Clinical studies for nursing students often emphasise observation-based training, offering limited hands-on experiences. This trend is not only due to the growing number of nursing education institutions and students but also because of increased patient rights and a heightened focus on patient safety [5]. Other challenges with the clinical field as a learning environment for nursing students include issues such as understaffing, heavy workloads and limited time for preceptors to effectively mentor students [6, 7]. Incorporating more simulation exercises into the nursing education could provide better control of the learning environment to improve nursing students’ satisfaction and ensure that they achieve the required competence levels [8, 9]. Simulation can be an effective educational method as it offers nursing students the opportunity to gain experience and learn about managing clinical cases in a safe environment at nursing education institutions [10, 11]. Simulation can be defined as ‘an educational strategy in which a particular set of conditions are created or replicated to resemble authentic situations that are possible in real life’ [12]. Furthermore, it is recommended that simulation exercises should consist of three phases: the briefing phase, the scenario phase and the debriefing phase [12]. The scenario phase provides participants with an opportunity to meet specified objectives within a simulated environment. Several published review studies have demonstrated that participation in simulation exercises boosts both the knowledge and confidence of undergraduate nursing students [11, 13–15]. In a Norwegian context, Olaussen et al. [16] examined knowledge acquisition and self-efficacy among first-year nursing students. An intervention group, which had 10.7% of their clinical placement hours in nursing homes replaced with simulation training, was compared to a control group following the traditional Norwegian model with clinical practice limited to nursing homes. Integrating the partial replacement of clinical hours in nursing homes with simulation training was positively associated with knowledge acquisition and meeting learning needs. These findings suggest that simulation could be a promising partial substitute for traditional clinical practice in nursing homes to enhance learning outcomes [16]. To increase the use of simulation exercises in nursing education, it is important that we have nurse educators who are trained to conduct simulations. Furthermore, research on simulation in undergraduate nursing education most often focuses on standard medical specialties and acute care in hospital settings, which underlines the need to explore simulation experiences in nonhospital settings within nursing education [17]. Therefore, the aim of this study was to identify, describe and discuss the factors that facilitate the successful implementation of simulation exercises for the transition to clinical practice in primary healthcare settings experienced by nursing students.

## Methods

### Design

This study employed a qualitative design and included seven focus group interviews. Focus groups are effective for capturing the diverse viewpoints and emotions that participants have regarding a topic, making them suitable for this study [18]. To explore nursing students’ experiences, Kirkpatrick’s four-level training evaluation model was applied. The model includes the following levels: reaction, learning, behaviour and results [19]. This study was conducted at level one (reaction), where participants assess the training by reflecting on their experiences with teaching and learning. This training evaluation highlights participants’ motivation and interest in learning, providing valuable insights for decision-making regarding the future development of the training programme [19]. The study adhered to the Consolidated Criteria for Reporting Qualitative Research (COREQ) checklist [20].

### Participants and settings

A convenience sample (*n* = 72) of undergraduate nursing students from two campuses within one university was invited to participate in the study. The first author contacted seven nurse educators employed at the university, who subsequently invited all their students in clinical practice to participate. To participate in the study, the students had to express interest to the first author. A total of 35 participants voluntarily responded and expressed their interest to participate in the study. No one who wished to participate was excluded. A total of seven focus groups were conducted: three with first-year students (*n* = 14; with 5, 3, and 6 participants in each), two with second-year students (*n* = 8; each with 4 participants), and two with third-year students (*n* = 13; two groups of 8 and 5 participants respectively). The groups were composed of students who were already classmates.

Two simulation scenarios were conducted at the educational institution before each practice period, covering clinical practice in nursing homes, home healthcare and psychiatric institutions in primary healthcare. The themes of the six scenarios were vital signs in acute deterioration, dementia, low blood sugar in diabetes, palliative care, suicide prevention and ethics (see Table 1). A patient simulator was used for one scenario, and a standardised patient or actor was used for the others. A standardised patient is ‘a person trained to consistently portray a patient or other individual in a scripted scenario for the purposes of instruction, practice, or evaluation’ [12]. Simulation group sizes ranged from 6 to 15 students, with 1–2 nurse educators operating the simulator, guiding students and sometimes playing the role of the patient (see Table 1).

**Table 1:** Information regarding the simulation exercises in the study

Simulation exercises before clinical practice in nursing homes (first-year students)	- The scenarios were about vital signs in acute deterioration and dementia. -A patient simulator was used in the scenario about vital signs in acute deterioration. - A student played the role of the patient in the scenario about dementia. - The simulation groups consisted of 6–8 members.
Simulation exercises before clinical practice in home healthcare (second-year students)	- The scenarios were about low blood sugar in diabetes and palliative care. - A standardised patient was used in the scenario of low blood sugar in diabetes. - A student played the role of the patient in the scenario about palliative care. - The simulation groups consisted of 13–15 members.
Simulation exercises before clinical practice in psychiatric institutions in primary healthcare (third-year students)	- The scenarios were about suicide prevention and ethics. - Standardised patients were used in both scenarios. - The simulation groups consisted of 6–8 members.

Following the International Nursing Association for Clinical Simulation and Learning’s (INACSL) Standards of Best Practice in Simulation [12], all simulation exercises were divided into three phases: brief, scenario and debrief. None of the exercises exceeded 90 min, with scenarios lasting no more than 15 min. The debriefing phase was structured according to Steinwachs’ model, which includes descriptive, analytic and application phases [21].

### Data collection

The aim of the study and literature formed the basis for the development of the interview guide. The self-developed interview guide consisting of the following five open-ended questions was used: (1) What role did you have in the simulation exercises?; (2) How would you describe your experience with the simulation exercises?; (3) What were the positive points of the simulation exercises?; (4) What were the negative points of the simulation exercises?; and (5) Do you have anything to add about the simulation exercises? Several follow-up questions, such as ‘Can you elaborate on that?’ and ‘What did you think of that?’, were asked. All interviews were audio-recorded and lasted between 16 and 27 min, with an average of 20 min. They were conducted by the first author in a classroom at the university where the simulation exercises took place. All the participants were physically present, and no interruptions occurred during the conduct of the interviews. In four of the interviews, a coauthor also participated. All interviews were conducted within one month after the final simulation exercise in the period from December 2021 to September 2022.

### Ethical perspectives

This study was conducted in accordance with the principles of the Declaration of Helsinki [22]. The participants were provided with verbal and written information concerning the study. The information included e.g. how their statements would be used, how their interviews and transcripts would be handled, and where the information would be stored. The students were also informed of their right to withdraw from the study at any time, and that participation or non-participation would not influence any aspect of their clinical placement or educational program. All students provided signed informed consent to participate. The research team members were not in a teacher-student relationship with the students before or during the study period. Participation in the simulation exercises was a compulsory part of nursing education. However, participating in the study were voluntary and did not affect students’ course grades. The participants did not receive any financial or non-financial benefits for participating in the study. Institutional approval from The Research Ethics Committee at Faculty of Health and Sport Sciences, University of Agder in Norway was granted to recruit the participants and perform the data collection. Additionally, the Norwegian Agency for Shared Services in Education and Research approved the study.

### Data analysis

The focus group interviews were transcribed verbatim by the first author. All words spoken in the focus groups were transcribed using the software program Microsoft Word. A reflexive thematic analysis using the following six steps was performed by the first author to identify codes, subthemes and themes: (1) familiarisation with the data; (2) coding; (3) initial theme generation; (4) reviewing and developing themes; (5) refining, defining and naming themes; and (6) producing the report [23]. In the first step, the transcribed text was read thoroughly by all the authors to ensure familiarisation with the data and identify meanings and patterns within them. Some patterns were discussed collaboratively. In the next step, the first author (KH) concentrated on identifying as many possible codes as possible. These codes were then discussed among all authors to refine the analysis and deepen the interpretation of the data. In the third step, themes were identified based on the initial codes, with some codes forming main themes and others forming subthemes. During the fourth step, the identified themes underwent review. In the fifth step, both themes and subthemes were defined and refined further. Subthemes represented elements within a larger main theme, providing structure to more complex themes. The final themes were reviewed and discussed by all the authors until a final agreement was reached. Finally, in the sixth step, the themes and subthemes were documented in relation to the data. Four examples of the reflexive thematic analysis are presented in Table 2.

**Table 2:** Examples of the reflexive thematic analysis resulting in four main themes

Examples of text coded	Subtheme	Theme
I thought it was very good that the groups were small because it didn’t feel like performing in front of a large audience (participant 5, focus group 6)	Composition of groups	Feeling secure
I think an ideal size for the simulation group is six people. That way, three can be in active roles, and three can be observers(participant 3, focus group 4)	Composition of groups	Feeling secure
And I found it more useful when we had a teacher act as a patient. As a student, you can’t always demonstrate everything (participant 4, focus group 1).	The patient role	Experiencing realism
I think it was a realistic situation. And for my part, I often find that if I experience such situations in clinical practice, I find it difficult to know what to do. And then it's nice to see how others handle it in the simulation exercise (participant 1, focus group 2).	Realistic situations	Experiencing realism
I think I gained more from it because I was in an active role. Instead of just sitting and observing (participant 4, focus group 2).	Active versus passive role	Learning in different roles
I found it very helpful to go through it together with the teacher at the end, where we gathered in a circle and reflected on the situation. I thought that it was very useful to hear different perspectives (participant 4, focus group 5).	Different perspectives	Reflecting together

### Rigour of the study

In qualitative analyses, aspects of trustworthiness such as credibility, dependability, transferability and confirmability are maintained through the chosen procedure for the qualitative design [24]. In this study, criteria for credibility, which relates to keeping the focus of the project, were met by choosing participants relevant for the aim of the study and including participants with various experiences to ensure shedding light on the research question from a variety of perspectives. Dependability, which relates to preventing inconsistency during data collection, was met by using the same interview guide for all participants. To facilitate transferability, we have provided a thorough description of the participants, context and research-process. Confirmability was improved through team discussions on coding and themes and by maintaining an audit trail of analytical decisions.

## Results

### The participants

The participants included 24 females and 11 males, ranging in age from 19 to 51 years (median: 22, average: 25). Eight participants had prior experience with simulation exercises before their nursing education (see Table 3). Twenty-four participants actively assumed roles as nurses, nursing students, or patients in the scenarios, while the remaining participants served as observers.

**Table 3:** Participant demographics (n = 35)

	Nursing homes(n = 14 first year students)	Home healthcare(n = 8 second year students)	Psychiatric institutions in primary healthcare (n= third year students)
*Gender*			
Female	11	5	8
Male	3	3	5
*Age in years*			
Md (Range)	20 (19–30)	21 (20–25)	25 (21–51)
*Previous experience with simulation before undergraduate nursing education*			
Yes	1		7
No	13	8	6

### Main themes

**Fig. 1:**
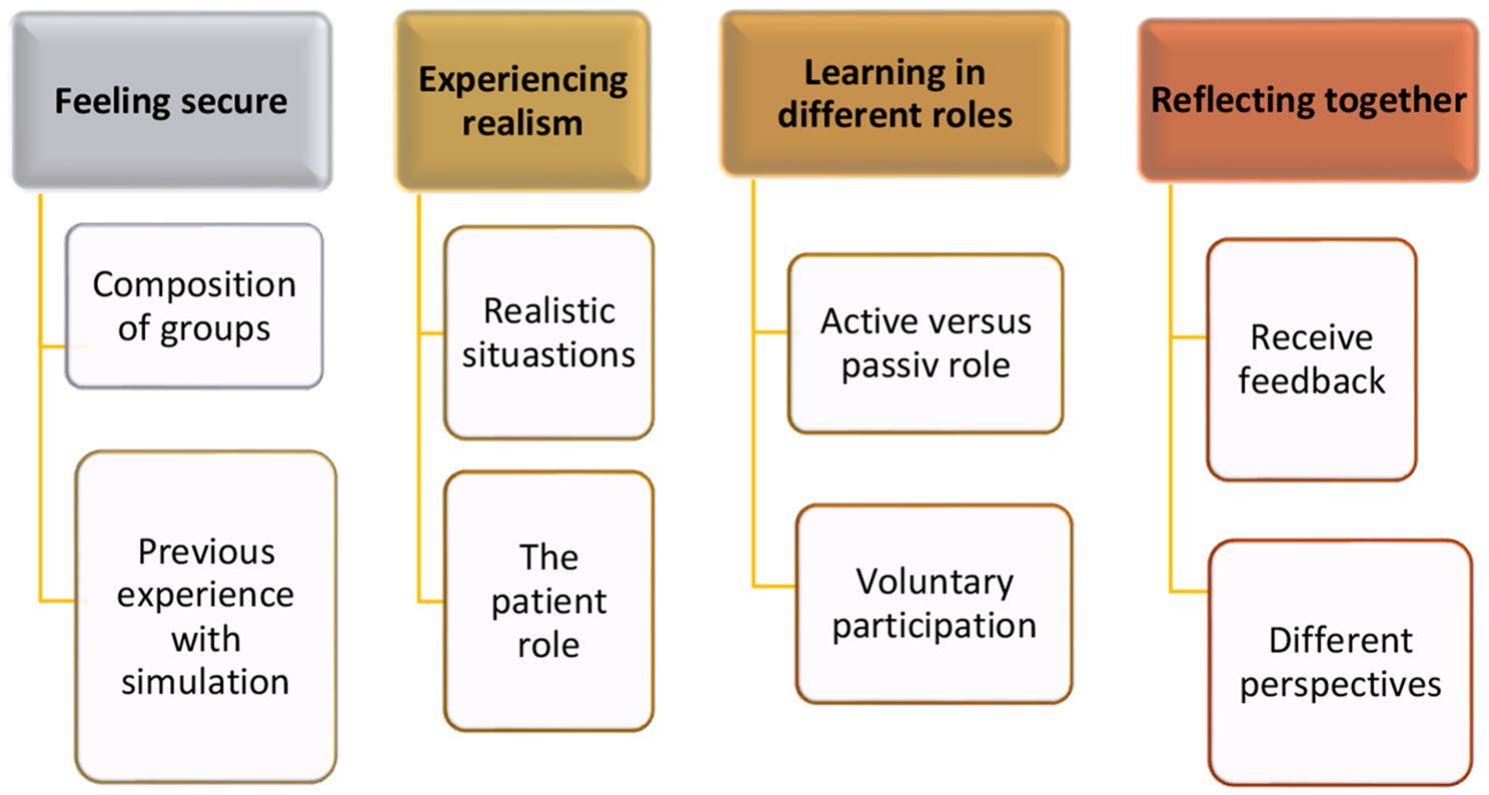
The four main themes and eight subthemes in the study

From the focus group interviews, four main themes were identified. These were interpreted as factors that facilitate the successful implementation of simulation exercises for transition to clinical practice in primary healthcare settings were identified: (a) feeling secure; (b) experiencing realism; (c) learning in different roles; and (d) reflecting together. Data extracts from the focus groups are included within the themes to illustrate their significance. The main themes and subthemes are presented in Fig. 1.

#### Feeling secure

Participants across all focus groups stressed that feeling secure in simulation exercises was crucial for learning. Familiarity with fellow students and teachers enhanced this secure feeling, and smaller groups were preferred. A third-year participant expressed it like this: *‘We were small groups now. It was very important to make you feel secure. You are not so afraid to make mistakes then’* (Participant 2, Focus Group 7). The proposed ideal group sizes were four to six students. Larger groups were perceived to have several disadvantages. A second-year participant said: *‘It was difficult to get to speak in a group with fifteen participants. There is also a lot of repetition because everything is said by several people’* (Participant 3, Focus Group 2). According to the participants, large groups increased anxiety, and the presence of many observers was perceived as stressful. A second-year participant said: *‘I think it was scary when there was a large audience’* (Participant 2, Focus Group 2). Some participants suggested to hide the observers a bit to make the participants feel more secure.

To be able to read the scenarios in advance was perceived by students to be useful to feeling prepared. This was expressed mainly by the first-year students. Conversely, participants with more experience with simulation expressed that they wanted more unexpected elements during the scenario. The fact that simulations were seen as practice rather than tests increased participants’ confidence during the exercises.

#### Experiencing realism

Participants emphasised the need for realistic and recognisable scenarios in preparing for clinical practice. A second-year participant said: *‘When a situation came up in practice that was quite like the one we had in the simulation*,* I thought: yes*,* we have experienced that before. So*,* then I knew what to do’* (Participant 2, Focus Group 2). Most participants felt simulations were valuable for clinical preparation. A first-year participant expressed it like this: *‘I think it’s a nice transition to clinical practice. It would have been a nice transition to each clinical practice we are going to have’* (Participant 3, Focus Group 4). In contrast to use of standardised patients, use of patient simulators with the voice of nurse educators was perceived less realistic. A first-year participant said: *‘It wasn’t a manikin that spoke*,* it was the teacher who spoke for the manikin. So*,* it became extra unnatural’* (Participant 4, Focus Group 5). As a result, many participants preferred real people as patients. A second-year participant said: *‘I think it was good that we used real people and not patient simulators. Now*,* there were real people who could talk’* (Participant 1, Focus Group 2). In particular, the use of nurse educators or professionals as patients was favoured for their realistic portrayal. A third-year participant expressed it like this: *‘We had one nurse from practice who came and played the patient. And it was very similar to how we see it out in practice now. It was easier to imagine it’* (Participant 2, Focus Group 7). Another third-year participant said: *‘It didn’t feel as awkward when you as a student didn’t have to pretend to be sick. Then you could take the role that was most natural’* (Participant 1, Focus Group 7).

#### Learning in different roles

Participants discussed the value of learning in different roles within simulation exercises, finding benefits in both active participation and observation. A first-year participant said:

I found it very useful, being able to try to handle the situation as a nurse, and the fact that someone else observes you and says things that you weren’t fully aware of or didn’t think about yourself because you don’t see yourself in a way. So, I think it was very useful! (Participant 3, Focus Group 5)

Several participants also emphasised the importance of being allowed to choose if they wanted to be in an active role. This was particularly expressed among second-year students who were in the largest simulation groups. Although being in an active role was perceived to be rewarding, it was also described as challenging. For example, several participants expressed discomfort with acting, particularly when being observed by other students and nurse educators. A second-year participant said: *‘And there are many who find it uncomfortable to stand in front of everyone. I also find it uncomfortable to be part of the scenario. You are afraid of doing something wrong’* (Participant 4, Focus Group 2). Despite these challenges, many argued that nursing often requires a form of ‘acting’, as patients are sensitive to body language. A second-year participant highlighted this aspect:

Because nursing is to a certain extent a form of ‘acting’. You have a lot of patients who read your facial expressions, your body language is very important. I became very aware of such things then. But you can’t become aware of these things if you don’t get through that wall of ‘awkwardness’ and manage to get rid of it in a way. (Participant 4, Focus Group 1)

In addition to active participation in simulation exercises, participants found the role as an observer beneficial. Observers were tasked with providing different perspectives in the debriefing phase, which helped deepen everyone’s understanding of the simulation experience.

### Reflecting together

Most participants emphasised the importance of reflecting together with other students to learn from simulation exercises. They highlighted the significance of reflection before and after the scenario. A third-year participant explained it like this: *‘I learned more from the briefing before and the debriefing afterwards’* (Participant 1, Focus Group 7). Almost all participants agreed that the debriefing phase after the simulation scenario was particularly crucial for learning. Two third-year participants said: *‘At least afterwards*,* it was very useful—when we went through everything that was good and what we could integrate into our practice’* (Participant 2, Focus Group 7) and *‘That’s kind of where the learning is*,* I feel!’* (Participant 1, Focus Group 7). Those who played active roles found receiving feedback from fellow students and nurse educators especially beneficial. Some of these participants found being in an active role so stressful that they struggled to recall details afterwards, making the reflection afterwards with others who had different perspectives particularly useful. A first-year participant expressed it like this: *‘But when more people listen*,* there are many perspectives*,* and then there is a good dialogue’* (Participant 2, Focus Group 4). Given the value of debriefing, participants expressed a desire for more debriefing discussions about real practice situations with their nurse preceptors in clinical studies. Many also wanted more simulation exercises integrated into their education before, during and after practice.

## Discussion

This study aimed to identify, describe and discuss the factors that facilitate the successful implementation of simulation exercises for the transition to clinical practice in primary healthcare settings experienced by nursing students. Based on the interviews, we found that feeling secure, experiencing realism, learning in different roles and reflecting together are important factors for learning in simulation exercises. These findings align with Kirkpatrick’s model [19], specifically at level 1, where participants’ reactions to their simulation experiences are investigated. These experiences seem essential and form the basis for further developing simulations that can enhance higher levels of learning within Kirkpatrick’s framework. This could be at level 3, which focuses on behavioral studies to assess whether participants are applying what they’ve learned (changes in behavior), or at level 4, which focuses on the results, determining whether the training has a positive impact, for example, on patient outcomes [19].

Feeling secure in the simulation setting has also been identified as an important factor for learning in other studies [25–28]. For example, uncertainty was identified as a main challenge in a study exploring the role of facilitators in clinical simulations, which included data from 218 written reflections, a focus group of 8 facilitators and semi-structured interviews with 21 participants [26]. It was found that one of the main purposes in the briefing phase is to establish a psychologically safe environment for the participants. Suggested activities include reviewing learning objectives, creating a ‘fictional contract’ with ground rules for the simulation session and orienting participants to the equipment, environment, simulator, roles, time allotment and scenario [12, 29]. Furthermore, tailoring support, consolidating knowledge, promoting respectful interactions and organising small groups are all critical elements in maintaining a safe learning environment [30–32]. In this study, several participants expressed feelings of insecurity due to the large simulation groups and being observed by their fellow students and nurse educators. It was suggested that the optimal group size should be approximately four to six members. Consistent with our findings, a systematic review and meta-analysis examining the impact of group size on learning outcomes in simulations among undergraduate nursing students found that groups of six or fewer students tend to be more effective at promoting knowledge and skills [33]. In addition, an integrative review recently identified factors that contribute to student insecurity, including unclear expectations, unfamiliarity with the environment, discomfort with being recorded, a lack of preparedness due to insufficient knowledge and skills, anxiety about mistakes in front of peers, being observed, team collaboration pressure and assessment fears [25].

In addition to feeling secure, experiencing realism was found to be an important factor. Research [34] has proposed that physical, psychological and environmental elements may be important for students to experience realism. Physical elements include equipment and related tools, while psychological elements encompass the motions, beliefs and self-awareness of participants. Environmental elements comprise participant and instructor motivation and goals, the group’s culture, the degree of openness and trust and participants’ modes of thinking [34]. In this study, several participants stated that playing the role of the patient in the scenario felt unrealistic. They stressed that the role should be filled by someone experienced, such as a teacher or clinical supervisor. That the role of the patient should be filled by someone experienced, is supported by the findings of Kaldheim et al. [29]. In the qualitative study of Kaldheim et al. [29], perioperative nursing students’ said that getting a role as an actor, for example, a surgeon, midwife or the voice of the simulator did not create a good learning process with subsequent learning outcomes. Several participants in our study also said that they preferred standardised patients over patient simulators, as they felt more realistic in the given scenarios within primary healthcare settings. In another study, nursing students felt that using a patient simulator made the simulation exercise more realistic [27]. The participants in Haddeland et al.’s study [27] highlighted the importance of an advanced patient simulator that allowed them to feel the patient’s pulse as well as the importance of ensuring that the person controlling the simulator was not visible in the scenario. This demonstrates the importance of nurse educators evaluating the best equipment to use in each scenario to allow students to experience realism and achieve the desired learning outcomes within the specific context. For example, a low-fidelity technical simulation using standardised patients can provoke a high degree of emotional fidelity [35].

Furthermore, the participants in our study highlighted the value of learning in various roles. They learned from both active participation and observation. The findings align with the results of other studies, which show that students in active roles highly value receiving feedback on their performance from peers who have observed them [27–29]. However, the participants in our study valued being allowed to choose if they wanted to be in an active role. Several participants reported feeling stressed and uncomfortable when acting in roles they did not identify with, such as the patient role. In another study [36], participants who took on active roles in simulation exercises exhibited higher stress levels, as indicated by both objective measures—such as heart rate and cortisol levels—and subjective perceptions, compared to those occupying observer roles. Although nursing educators need to facilitate safety and allow students to choose whether they want to participate actively in simulation exercises, they should make sure that in doing so, they do not hinder the students’ learning. One study has emphasised that an optimal level of stress could sharpen students’ focus, improve their learning outcomes and enable them to gain clinical experience [37]. Research has also shown that educational development often takes place outside of one’s comfort zone; thus, by not offering challenging experiences, educators might unintentionally restrict students’ readiness for the complexities they will face in clinical practice [25]. Supplemental ways of offering challenging experiences and learning include the use of 360° video technology [38] and interactive computer-based simulations [39], which offer recordings from real practice environments. The observational and reflective possibilities of 360° videos make them particularly suitable for training nontechnical skills, such as social and interpersonal skills [38] and behavioural responses to distressed patients [40]. For example, 360° videos can be particularly useful to moderating anxiety and building confidence among nursing students before entering clinical placement in mental health nursing [40]. However, students will most likely also meet distressed patients in home healthcare and nursing homes. Combining virtual reality with traditional simulation in undergraduate nursing education not only offers students the chance to practice different roles (taking an active or passive role) but also grants them independent, repeatable practice time.

The fourth main theme identified in this study was the importance of reflecting together with other students to learn from simulation exercises. The participants said that the debriefing phase was particularly crucial for learning. Simulation debriefing has been characterised in numerous studies as an essential component of the simulation process employed for learning in nursing education [10, 41, 42]. The debriefing phase is defined as ‘a reflective process immediately following the simulation-based experience that is led by a trained facilitator using an evidence-based debriefing model’ [12]. In our study, not all nurse educators employed at the university were trained in organising simulation exercises. Nonetheless, they all conducted the debriefing phase using Steinwachs’ model, which encompasses descriptive, analytic and application phases [21]. A variety of debriefing techniques have been utilised in nursing education simulations to enhance clinical competencies and improve learning outcomes [43]. However, it has been noted that educators frequently overlook or disregard participants’ emotions, such as anger, frustration and anxiety, during debriefings [44]. To address this, it has been argued that the debriefing phase should begin with a reaction phase before progressing to the descriptive, analytic and application phases. By doing so, educators can mitigate the risk of leaving unresolved negative emotions that could potentially diminish the learning outcomes among participants [44].

### Strengths and limitations

There were some strengths and limitations to the present study. First, a strength of this study is that all the four female authors are academics with expertise in qualitative analysis. Second, the same interview guide was used for all participants and the same researcher conducted, transcribed and analysed all of the interviews (KH). Using the same interview guide for all participants ensures consistency in how data is collected. It guarantees that all participants are asked the same questions in the same manner, minimizing variability that could arise from different interview questions. Having the same researcher conduct, transcribe, and analyse all interviews further strengthens the study by maintaining uniformity and reducing potential biases. An additional strength of this study is that the findings are supported by quotes from first-, second- and third-year students from the undergraduate nursing program, providing rich data regarding their experience participating in simulations within nursing education. However, this might also be a study limitation. The group was not homogenous, so differences in experience with simulation might have affected their perceptions. The study included six different simulation scenarios that were conducted with various nurse educators, and there was also variation in the use of the patient role in the scenarios, with patient simulators, standardized patients, and actors being used. The size of the simulation groups varied as well. The total number of first- and third-year participants were similar in size (14 and 13, respectively) while only eight second-year students participated. There may be a self-selection bias, in that those who participated in the study may have been particularly motivated students or those with more positive experiences of simulation. Consideration must also be given in relation to transferability and the fact that the study has taken place in a Norwegian context. Since other countries may have different educational simulation practices and cultural contexts, this can affect the applicability of the study’s findings to other countries. However, our findings are supported by national and international research and theory. Thus, we argue that the findings of this study may be transferable to comparable contexts in relation to educational practices and simulation strategies.

## Conclusions

In this study, undergraduate nursing students pointed out key factors that facilitate successful simulation exercises for transitioning to clinical practice in primary healthcare settings. These factors were feeling secure, experiencing realism, learning in different roles and reflecting together. Furthermore, this study revealed that students encountered various elements in the organisation of each simulation exercise, such as group size, the use of simulators, standardised patients and taking on the role of the patient themselves. Thus, our study can fill a gap in the literature on simulation experiences in nonhospital settings and contributes to valuable insights that may guide future implementation of simulation exercises in undergraduate nursing education. With the increasing number of nursing education institutions, enhanced patient rights and a strengthened focus on patient safety, nursing students face challenges in practicing skills within clinical settings. Simulation proves to be an effective educational method, allowing nursing students to gain experience and learn how to manage clinical cases safely at nursing education institutions. Overall, the study underscores the importance of each educational institution developing a comprehensive programme for all simulation exercises in its curriculum that adheres to consistent quality indicators.

## Data Availability

The datasets generated and analysed in the present study are not publicly available due to concerns that participants’ privacy may be compromised; however, they are available from the corresponding author upon reasonable request.
